# Longitudinal Study of the Decline in Renal Function in Healthy Subjects

**DOI:** 10.1371/journal.pone.0129036

**Published:** 2015-06-10

**Authors:** Mika Baba, Takuro Shimbo, Masaru Horio, Masahiko Ando, Yoshinari Yasuda, Yasuhiro Komatsu, Katsunori Masuda, Seiichi Matsuo, Shoichi Maruyama

**Affiliations:** 1 Center for Preventive Medicine, St. Luke’s Affiliated Clinic, St. Luke’s International University, Tokyo, Japan; 2 Ohta Nishinouchi Hospital, Koriyama, Japan; 3 Department of Functional Diagnostic Science, Osaka University Graduate School of Medicine, Suita, Osaka, Japan; 4 Center for Advanced Medicine and Clinical Research, Nagoya University Hospital, Nagoya, Japan; 5 Department of Nephrology, Nagoya University Graduate School of Medicine, Nagoya, Japan; 6 Division of Nephrology, Department of Medicine, St. Luke’s Hospital, St. Luke’s International University, Tokyo, Japan; Tokushima University Graduate School, JAPAN

## Abstract

**Background:**

Chronic kidney disease is an important concern in preventive medicine, but the rate of decline in renal function in healthy population is not well defined. The purpose of this study was to determine reference values for the estimated glomerular filtration rate (eGFR) and rate of decline of eGFR in healthy subjects and to evaluate factors associated with this decline using a large cohort in Japan.

**Methods:**

Retrospective cross-sectional and longitudinal studies were performed with healthy subjects aged ≥18 years old who received a medical checkup. Reference values for eGFR were obtained using a nonparametric method and those for decline of eGFR were calculated by mixed model analysis. Relationships of eGFR decline rate with baseline variables were examined using a linear least-squares method.

**Results:**

In the cross-sectional study, reference values for eGFR were obtained by gender and age in 72,521 healthy subjects. The mean (±SD) eGFR was 83.7±14.7ml/min/1.73m^2^. In the longitudinal study, reference values for eGFR decline rate were obtained by gender, age, and renal stage in 45,586 healthy subjects. In the same renal stage, there was little difference in the rate of decline regardless of age. The decline in eGFR depended on the renal stage and was strongly related to baseline eGFR, with a faster decline with a higher baseline eGFR and a slower decline with a lower baseline eGFR. The mean (±SD) eGFR decline rate was ‒1.07±0.42ml/min/1.73m^2^/year (‒1.29±0.41%/year) in subjects with a mean eGFR of 81.5±11.6ml/min/1.73m^2^.

**Conclusions:**

The present study clarified for the first time the reference values for the rate of eGFR decline stratified by gender, age, and renal stage in healthy subjects. The rate of eGFR decline depended mainly on baseline eGFR, but not on age, with a slower decline with a lower baseline eGFR.

## Introduction

The number of patients with chronic kidney disease (CKD) has shown a continuous increase and renal impairment increases the risk of death and cardiovascular diseases [1,2]. Preventive measures for CKD have been promoted, including publication of the new Kidney Disease Improving Global Outcomes (KDIGO) CKD classification in 2011, in which it was proposed that the stage of renal function and level of proteinuria are predictive factors for subsequent renal function and prognosis, and that the risk is reflected by the color intensity in a heat map [3]. A faster rate of decline of renal function may cause death and has an increased risk of cardiovascular diseases [4–7]. This rate of decline may be a predictive factor [8] and has also been used as a criterion for evaluating the efficacy of renoprotective agents. In addition to increases in the serum creatinine level of 1.5- and 2-fold, a 40% or 30% decline in the estimated glomerular filtration rate (eGFR) from baseline have recently been proposed as new surrogate endpoints [9–11].

Renal impairment occurs with age. Therefore, both disease and aging affect renal function and it is useful to understand the change of renal function in aging process [12,13]. In a cross-sectional study in Japan, Okada et al. used the age- and gender-specific 5th and 95th percentiles for eGFR in healthy subjects to define the range of prediabetes mellitus and prehypertension [14]. In a longitudinal study, Imai et al. examined eGFR decline in healthy subjects and patients with hypertension and/or proteinuria [15]. However, the detailed eGFR distribution and rate of eGFR decline in healthy subjects have yet to be clarified.

The current work includes a cross-sectional study to determine reference values for eGFR stratified by gender and age group; and a longitudinal study of reference values for eGFR decline rate stratified by gender, age and renal stage in healthy subjects using a large Japanese cohort. Factors related to the rate of eGFR decline were also examined.

## Methods

### Data Collection

**Fig 1:**
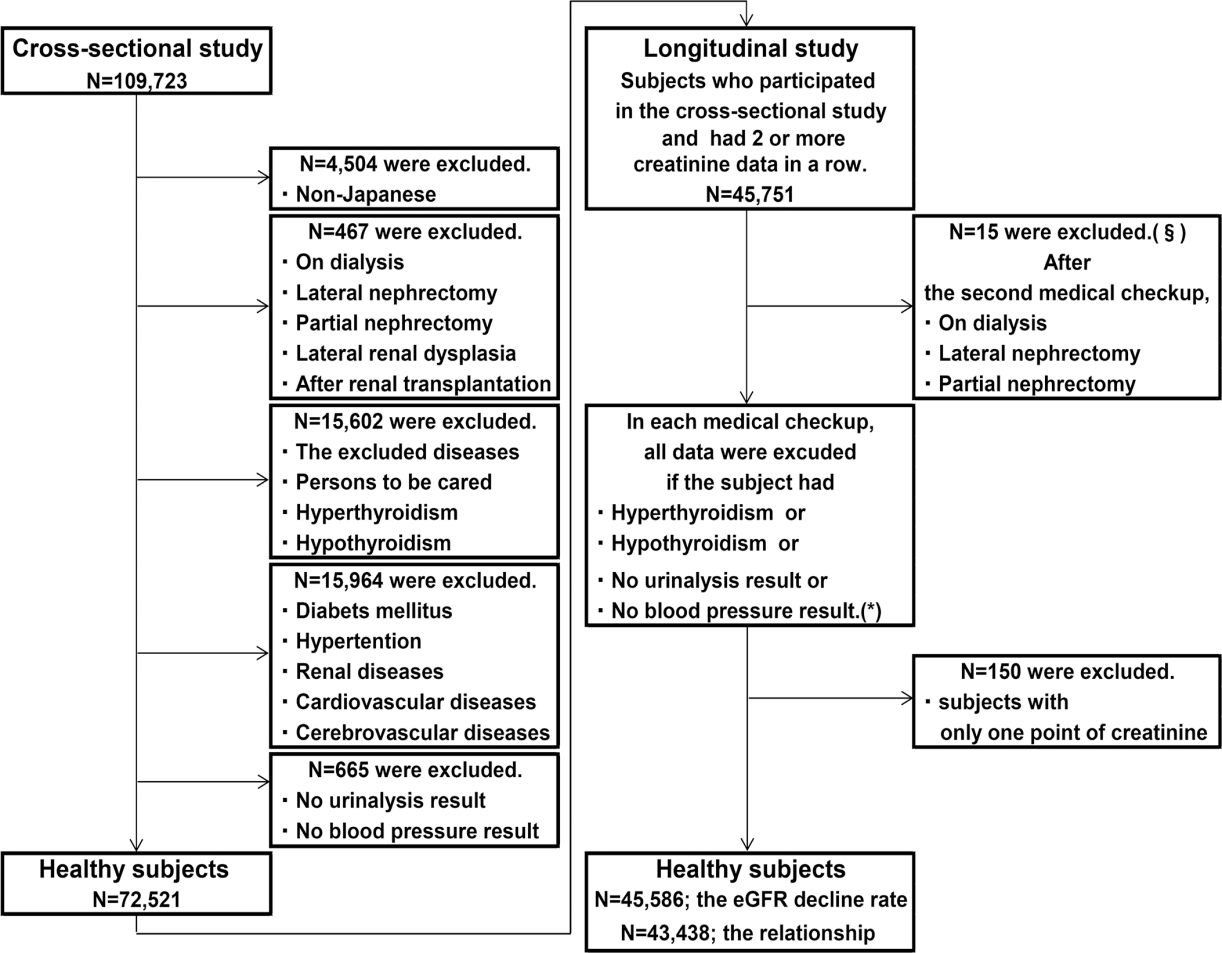
Flow diagram of healthy subjects. Persons without any diseases were defined as healthy subjects. There were 72,521 subjects in the cross-sectional study. In the longitudinal study, eGFR decline rate was evaluated in 45,586 subjects and relationships between eGFR decline rate and 15 baseline factors were evaluated in 43,438 subjects. ^§^After the second medical checkup, there was no subject who received renal transplantation. All data corresponding to the particular medical checkup were excluded for subjects diagnosed with hyperthyroidism or hypothyroidism based on blood tests or those without urinalysis or blood pressure measurements in a second or later medical checkup, and *the numbers of subjects for whom data were not included in each checkup were 567(2^nd^), 389(3^rd^), 277(4^th^), 216(5^th^), 153(6^th^), 99(7^th^), 64(8^th^), 37(9^th^), 5(10^th^), and 1(12^th^).

Retrospective cross-sectional and longitudinal studies were performed in people aged ≥18 years old who received a medical checkup at The Center for Preventive Medicine, St. Luke’s Affiliated Clinic, from January 2004 to December 2012. The study was conducted using a linkable anonymous dataset. No informed consent was obtained. The study protocol and consent procedure were approved by the ethics committee of St. Luke’s International University (Number: 12-R123). People who requested that their personal information should not be used when they received a medical checkup were excluded at the time the medical records were extracted. Information including medical history and examination data was extracted from electronic medical records. People without any diseases at baseline were defined as healthy subjects according to the flow diagram (Fig 1) described below [16].

### Flow Diagram

The goal of the study was to evaluate renal function in healthy Japanese subjects, and therefore the following individuals were excluded: 1) non-Japanese people identified by nationality and name; 2) those who were on dialysis or had undergone renal transplantation or nephrectomy (including lateral nephrectomy, donor nephrectomy, and partial nephrectomy), or those with a history of renal hypoplasia confirmed in medical records or abdominal echo findings at medical checkup; 3) those with the diseases listed in Table 1; 4) those with gait disturbance (who used crutches, a wheelchair, or a walking device due to neurological, muscular, or bone lesions), severe visual impairment, or severe cognitive impairment; 5) those diagnosed with hyperthyroidism (TSH ≤0.1 μU/ml and fT4 >1.64 ng/dl) or hypothyroidism (TSH ≥6.0 μU/ml and fT4 <1.00 ng/dl) based on blood tests at the medical checkup; 6) those with diabetes mellitus or impaired glucose tolerance (receiving drug therapy based on interview, HbA1c (Japan Diabetes Society) ≥6.1%, or fasting blood glucose ≥126 mg/dl in blood tests at the medical checkup); 7) those diagnosed with hypertension (receiving drug therapy based on interview, systolic blood pressure (SBP) ≥140 mmHg, or diastolic blood pressure (DBP) ≥90 mmHg at the medical checkup); 8) those diagnosed with renal diseases (under treatment for or a history of nephritis, nephrotic syndrome, or chronic renal failure based on interview, or urinary protein of 1+ and higher at the medical checkup); 9) those diagnosed with cardiovascular or cerebrovascular diseases (under treatment for or a history of angina, myocardial infarction, aortic aneurysm, cerebral infarction, intracerebral hemorrhage, or subarachnoid hemorrhage based on interview); and 10) those without data for urinalysis or BP (if not already excluded in items 1) to 9)).

**Table 1:** List of excluded diseases.

Malignant disease	Benign disease
**esophageal cancer**	**tuberculosis,pleuritis**
**stomach cancer**	**atypical mycobacterial disease**
**colon cancer**	**chronic hepatitis B**
**liver cancer**	**chronic hepatitis C**
**lung cancer**	**blood disease except anemia**
**breast cancer**	**hypothyroidism**
**cervical cancer**	**hyperthyroidism**
**uterine cancer**	**thyroid disease**
**ovarian tumor**	**Parkinson's disease**
**prostate cancer**	**valvular disease**
**brain tumor**	**chronic bronchitis**
**other cancer**	**pulmonary emphysema**
	**chronic obstructive pulmonary disease**
	**bronchiectasis**
	**interstitial pneumonia**
	**pulmonary fibrosis**
	**Crohn's disease**
	**ulcerative colitis**
	**liver cirrhosis**
	**chronic pancreatitis**
	**collagen disease**
	**kidney stone**
	**ureteral stone**
	**ventricular septal defect**
	**atrial septal defect**

The candidates were judged based on exclusion criteria 1) to 10) and registered as subjects of the cross-sectional study. The same subjects were candidates for the longitudinal study, but this study required serial data. Therefore, subjects with the following conditions were excluded: 11) those with only one serum creatinine value and 12) those with a history of dialysis or after renal transplantation or nephrectomy (including lateral nephrectomy, donor nephrectomy, and partial nephrectomy) that was newly revealed by medical records or abdominal echo findings at a second or later medical checkup. In the case of the following conditions, all data corresponding to the particular medical checkup were excluded for the subject: 13) hyperthyroidism (TSH ≤0.1 μU/ml and fT4 >1.64 ng/dl) or hypothyroidism (TSH ≥6.0 μU/ml and fT4 <1.00 ng/dl) based on blood tests at a second or later medical checkup, and 14) no data for urinalysis or BP. After examining the candidates for the longitudinal study based on criteria 1) to 14), those with only one serum creatinine value were excluded. Active hyperthyroidism decreases the creatinine level, active hypothyroidism increases this level, and creatinine returns to the original level as the hormone level returns to the normal range [17,18]. Thus, patients with active hyperthyroidism or hypothyroidism were excluded due to the transient variation of creatinine.

### Estimation of eGFR

After an overnight fast, subjects received a medical checkup in the morning. Blood tests were performed in the Laboratory of the Center for Preventive Medicine, St. Luke’s Affiliated Clinic, on the day of the medical checkup. Serum creatinine was measured with an enzymatic method on an autoanalyzer (Bio Majesty JCA-BM, Jeol Ltd. Tokyo, Japan), which data were rounded to the second decimal point. eGFR was calculated using the 3-variable Japanese equation [19]: eGFR (ml/min/1.73m^2^) = 194 × sCr^‒1.094^ × Age^-0.287^ (if female × 0.739)

### Statistical Analysis

The cross-sectional study was performed using data from the first medical checkup. Reference values for eGFR (mean and 2.5th, 5th, 25th, 50th, 75th, 95th, and 97.5th percentiles) stratified by gender and age in 5-year increments were evaluated using a nonparametric method [16]. The change in eGFR with older age (herein defined as the “generation change”) was calculated as the coefficient of the fitted line estimated for the relationship between eGFR and age by linear least-squares regression analysis.

The rate of eGFR decline was determined in units of “ml/min/1.73m^2^/year” (rate defined as the “slope”) and “%/year” (rate defined as the “%slope”). In the longitudinal study 1, the individual rate of eGFR decline (i.e. the slope; ml/min/1.73m^2^/year) was calculated using a mixed model analysis in which random-intercept and random-slope were fitted. In this analysis, the dependent variable was eGFR, the independent variable was days after the first visit, and then the slope was obtained by multiplying 365.25. In the longitudinal study 2, the individual slope was divided by the individual eGFR at baseline and this value was defined as the %slope ([ml/min/1.73m^2^/year] / [intercept of eGFR] ×100 in unit of %/year). The individual slope and the individual %slope were stratified by gender, age group and renal stage, and the means of the slope and the %slope were calculated in each stratified component. To minimize the effect of a “regression to the mean”, the individual intercept of eGFR was used as the baseline eGFR when the individual slope (longitudinal study 1) and the individual %slope (longitudinal study 2) were stratified to the renal stage. Renal function was classified into KDIGO stages G1 to G3b. Since the study included healthy subjects with generally higher eGFR, stages G1 and G2 were divided into 2 categories by 15 ml/min/1.73m^2^. There were no subjects in stages G4 and G5 in the study.

In the longitudinal study 3, the Relationships of eGFR decline rate with 15 factors at baseline (intercept of eGFR, age, gender, urinary protein by dipstick test, serum uric acid, HbA1c, fasting blood glucose, SBP, serum phosphorus, serum calcium, HDL-cholesterol, non-HDL-cholesterol, triglycerides (TG), BMI, and smoking status) was evaluated using a linear least-squares regression analysis. Subjects who lacked any of these data were excluded, and the number of subjects in this analysis decreased from 45,586 to 43,438. There were high correlations between SBP and DBP, non-HDL- and total cholesterol, and non-HDL- and LDL-cholesterol (correlation coefficients of 0.86, 0.90, and 0.94, respectively), and thus SBP and non-HDL-cholesterol were selected as factors because these parameters are most frequently used in clinical settings. Log-transformed TG was used because the distribution of this data was skewed. P<0.0033 (0.05/15 = 0.0033) using the Bonferroni correction was considered to be significant. STATA software (ver. 13.1) was used for the analysis.

### Graph of the Slope of eGFR Decline (Simulated Change of eGFR)

The slope of the rate of eGFR decline was depicted as the inclination of each line and the trajectory of renal function was predicted by simulation [15]. Each long line of the graph was not an individual's trajectory, but a connected trajectory using the mean slope of eGFR decline in stratified component.

## Results

### Characteristics

There were 72,521 subjects in the cross-sectional study and 45,586 subjects in the longitudinal study. Characteristics in the longitudinal study were based on laboratory data at baseline (Table 2). The intercept of eGFR was used as the baseline value of eGFR. The mean (±SD) ages of the subjects in the cross-sectional and longitudinal studies were 42.8±10.4 and 43.9±10.2 years, respectively, and the ratios of men to women were 47% and 48%, respectively. The mean (±SD) eGFR was 83.7±14.7 ml/min/1.73m^2^ in the cross-sectional study and the mean (±SD) baseline eGFR was 81.5±11.6 ml/min/1.73m^2^ in the longitudinal study. Other parameters were within normal ranges. Creatinine was measured once in the cross-sectional study and 2 to 18 times in a row in the longitudinal study, with a median of 4 times for men and women (S1 Fig). The mean (±SD) intervals between the first and last medical checkups were 4.19±2.45 years in men and 4.35±2.47 years in women (S2 Fig).

**Table 2:** Characteristics of healthy subjects in the cross-sectional and longitudinal studies.

	Cross-sectional study		Longitudinal study	
Characteristics	mean±SD or N(%)	N	mean±SD or N(%)	N
**Age(y)**	**42.8±10.4**	**72521**	**43.9±10.2**	**45586**
**Men gender**	**33988 (47%)**	**72521**	**21703 (48%)**	**45586**
**Serum creatinine(mg/dl)**	**0.73±0.15**	**72521**	**0.72±0.15**	**45586**
**eGFR (ml/min/1.73m** ^**2**^ **)**	**83.7±14.7**	**72521**	**81.5±11.6** §	**45586**
**Urine protein**		**72521**		**45586**
**Negative(-)**	**69554 (96%)**		**44068 (97%)**	
**Trace(15mg/dl;±)**	**2967 (4%)**		**1518 (3%)**	
**Uric acid(mg/dl)**	**5.19±1.40**	**72521**	**5.20±1.40**	**45586**
**HbA1c(%)**	**4.99±0.32**	**71459**	**4.99±0.33**	**44869**
**Fasting blood glucose(mg/dl)**	**96.1±8.0**	**72521**	**96.4±8.0**	**45586**
**Systolic blood pressure(mmHg)**	**112.4±12.8**	**72521**	**112.5±12.8**	**45586**
**Serum phosphorus(mg/dl)**	**3.46±0.42**	**70457**	**3.45±0.42**	**44155**
**Serum calcium(mg/dl)**	**9.21±0.33**	**70456**	**9.20±0.32**	**44155**
**HDL-cholesterol(mg/dl)**	**63.1±15.4**	**72521**	**63.2±15.5**	**45586**
**NonHDL-cholesterol(mg/dl)**	**134.8±34.8**	**72006**	**136.1±34.7**	**45371**
**Triglycerides(mg/dl)** *	**73 [52, 109]** *	**72521**	**74 [52, 111]** *	**45586**
**Body mass index**	**21.9±3.0**	**72520**	**22.0±3.0**	**45586**
**Smoking**		**72521**		**45586**
**Never-smoked**	**44252 (61%)**		**27966 (61%)**	
**Ex-smoker**	**14314 (20%)**		**9102 (20%)**	
**Current smoker**	**13955 (19%)**		**8518 (19%)**	

**Note**: § intercept of eGFR. interquartile range.

### Reference Values for eGFR by Gender and Age (Cross-Sectional Study)

**Fig 2:**
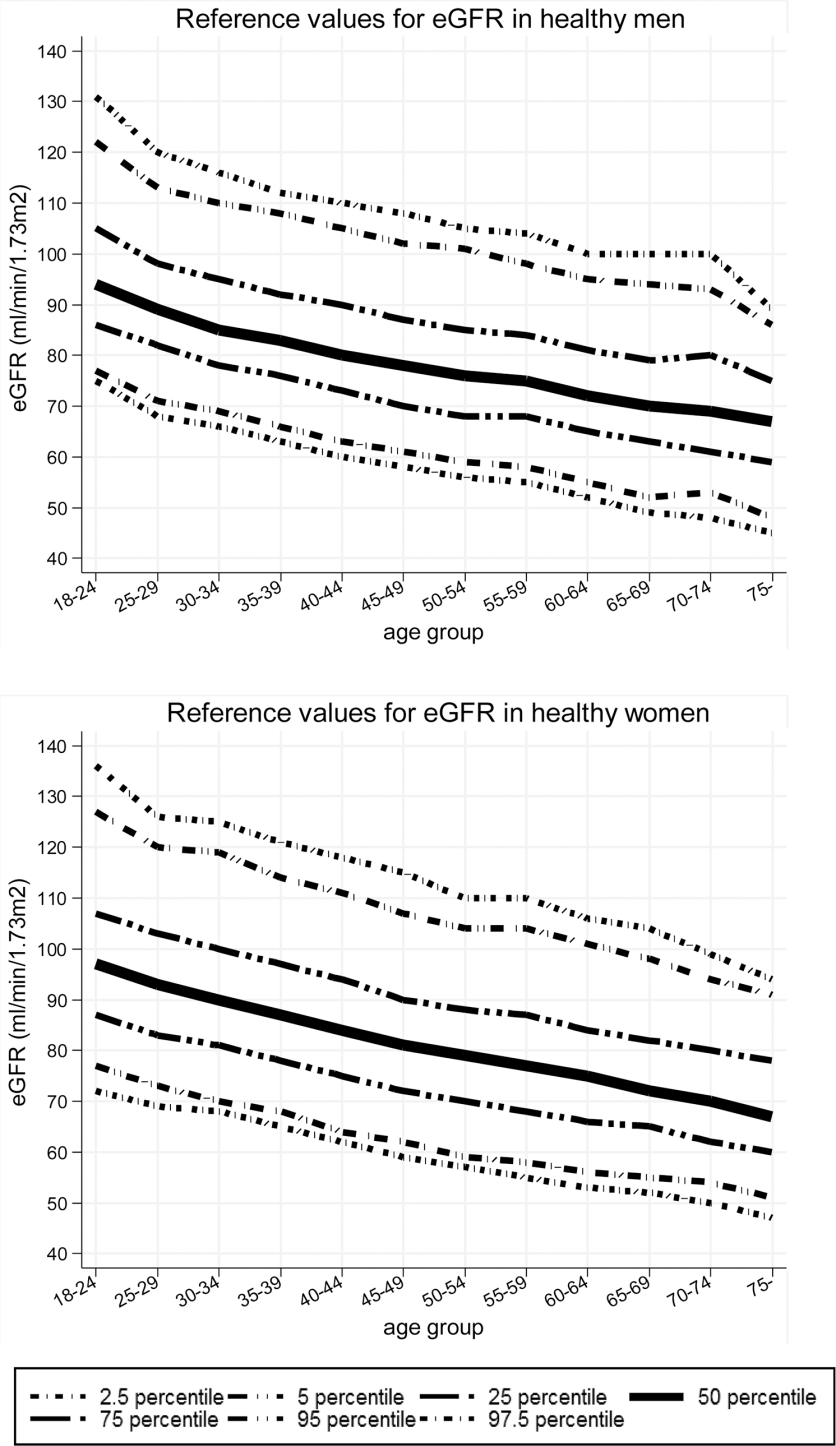
Reference values for eGFR in healthy men and women in the cross-sectional study. Values for eGFR decreased with older age in both genders. eGFR was calculated using the 3-variable Japanese equation.

Reference values were obtained for eGFR stratified by gender and age (mean and 2.5th, 5th, 25th, 50th, 75th, 95th, and 97.5th percentiles; Fig 2 and S1 Table). Values for eGFR decreased with older age in both genders. The generation changes were ‒0.51 ml/min/1.73m^2^/year in all subjects, ‒0.46 ml/min/1.73m^2^/year in men and ‒0.54 ml/min/1.73m^2^/year in women (S3 Fig). The 2.5th percentiles of eGFR at age ≥50 years old were ≤56 (in men) and ≤57 (in women), and 5th percentiles were ≤59 ml/min/1.73m^2^ in both genders (Fig 2 and S1 Table).

### Reference Values for the Slope of eGFR Decline by Gender, Age, and Renal Stage (Longitudinal Study 1)

Reference values for the slope of eGFR decline stratified by gender, age, and renal stage were determined in the longitudinal study 1(Tables 3 and 4). In the same renal stage, there was little difference in the slope of eGFR decline at different ages. For example, in men (Table 3), the slope ranged from ‒1.02 to ‒1.12 ml/min/1.73m^2^/year and the mean slope was ‒1.06 ml/min/1.73m^2^/year for baseline eGFR 75 to 89 ml/min/1.73m^2^. A similar tendency was seen in women (Table 4). In contrast, in the same age group, the slope was steeper when baseline eGFR was higher (i.e. better renal function) and shallower when baseline eGFR was lower (i.e. renal stage changed from G1 to G3b). For example, in men aged 40 to 44 years (Table 3), the slopes were ‒1.81 (eGFR ≥105), ‒1.44 (eGFR 90–104), ‒1.07 (eGFR 75–89), ‒0.78 (eGFR 60–74), and ‒0.46 ml/min/1.73m^2^/year (eGFR 45–59). The same tendency was observed in each age group in men and women (Table 3 and Table 4). With stratification by gender and age, but not by renal stage, the slope of the eGFR decline appeared to be shallower with aging. For example, in men (Table 3), the slope ranged from ‒1.38 (age 18–24) to ‒0.83 ml/min/1.73m^2^/year (age ≥75) for baseline eGFR of 30≤. This may have been influenced by lowering of the mean eGFR with aging (93.67 at age 18–24 to 66.46 ml/min/1.73m^2^ at age ≥75), as also seen in women (Table 4). To present the distribution of the slope in each component, the mean slope ±2SD are shown in S2 and S3 Tables. The mean (±SD) slopes in the longitudinal study were ‒1.07±0.42 ml/min/1.73m^2^/year in all subjects, ‒1.03±0.40 ml/min/1.73m^2^/year in men, and ‒1.11±0.44 ml/min/1.73m^2^/year in women.

**Table 3:** Slope (ml/min/1.73m^2^/year) of eGFR decline in healthy men.

Men			Age												
Stage	Intercept of eGFR	Slope	18–24	25–29	30–34	35–39	40–44	45–49	50–54	55–59	60–64	65–69	70–74	75≤	18≤
**G1**	**105≤**	**ml/min/1.73m** ^**2**^ **/year**	**-1.94**	**-1.92**	**-1.78**	**-1.79**	**-1.81**	**-1.80**	**-1.73**	**-1.68**	**-1.99**	**-2.24**	**-1.34**		**-1.81**
	mean of intercept		111.57	111.63	110.08	110.48	111.23	111.01	110.12	111.14	110.03	108.05	113.98		110.74
		N	17	48	88	120	65	36	24	16	4	2	2	0	422
	**90–104**	**ml/min/1.73m** ^**2**^ **/year**	**-1.42**	**-1.38**	**-1.40**	**-1.44**	**-1.44**	**-1.38**	**-1.42**	**-1.42**	**-1.54**	**-1.37**	**-1.18**		**-1.42**
	mean of intercept		95.47	96.02	95.51	95.17	95.15	95.09	94.81	94.51	95.63	95.23	96.08		95.27
		N	54	309	679	949	611	299	208	128	48	21	10	0	3316
**G2**	**75–89**	**ml/min/1.73m** ^**2**^ **/year**	**-1.12**	**-1.05**	**-1.05**	**-1.08**	**-1.07**	**-1.06**	**-1.04**	**-1.06**	**-1.04**	**-1.02**	**-1.12**	**-1.03**	**-1.06**
	mean of intercept		84.54	83.41	82.66	82.41	81.78	81.50	81.00	81.00	80.49	80.24	81.58	80.08	81.91
		N	44	464	1497	2700	2334	1384	918	772	390	150	49	14	10716
	**60–74**	**ml/min/1.73m** ^**2**^ **/year**		**-0.79**	**-0.78**	**-0.79**	**-0.78**	**-0.76**	**-0.76**	**-0.76**	**-0.74**	**-0.78**	**-0.78**	**-0.87**	**-0.77**
	mean of intercept			71.89	71.05	70.70	70.08	69.59	69.13	68.88	67.98	67.52	68.08	67.84	69.48
		N	0	43	301	946	1296	1124	1048	897	613	292	117	47	6724
**G3a**	**45–59**	**ml/min/1.73m** ^**2**^ **/year**			**-0.49**	**-0.46**	**-0.46**	**-0.53**	**-0.51**	**-0.48**	**-0.50**	**-0.57**	**-0.58**	**-0.62**	**-0.52**
	mean of intercept				59.90	57.72	56.47	57.21	57.22	56.73	56.37	55.84	55.57	54.85	56.51
		N	0	0	1	10	26	64	89	82	108	86	37	20	523
**G3b**	**30–44**	**ml/min/1.73m** ^**2**^ **/year**										**-0.09**		**-0.01**	**-0.05**
	mean of intercept											38.36		42.82	40.59
		N	0	0	0	0	0	0	0	0	0	1	0	1	2
**G1-G3b**	**30≤**	**ml/min/1.73m** ^**2**^ **/year**	**-1.38**	**-1.20**	**-1.14**	**-1.11**	**-1.05**	**-0.97**	**-0.93**	**-0.92**	**-0.86**	**-0.84**	**-0.85**	**-0.83**	**-1.03**
	mean of intercept		93.67	88.92	85.63	83.29	80.45	78.13	76.19	75.38	72.38	70.30	70.73	66.46	80.05
		N	115	864	2566	4725	4332	2907	2287	1895	1163	552	215	82	21703

**Table 4:** Slope (ml/min/1.73m^2^/year) of eGFR decline in healthy women.

Women			Age												
Stage	Intercept of eGFR	Slope	18–24	25–29	30–34	35–39	40–44	45–49	50–54	55–59	60–64	65–69	70–74	75≤	18≤
**G1**	**105≤**	**ml/min/1.73m** ^**2**^ **/year**	**-1.54**	**-1.78**	**-1.85**	**-1.88**	**-1.96**	**-1.96**	**-1.83**	**-1.90**	**-2.02**	**-1.50**		**-1.91**	**-1.87**
	mean of intercept		115.68	111.78	111.60	111.14	111.30	110.73	109.58	111.91	110.25	123.24		106.55	111.36
		N	15	95	311	329	158	68	32	16	9	1	0	1	1035
	**90–104**	**ml/min/1.73m** ^**2**^ **/year**	**-1.45**	**-1.36**	**-1.39**	**-1.42**	**-1.42**	**-1.51**	**-1.44**	**-1.50**	**-1.37**	**-1.40**	**-1.58**	**-1.51**	**-1.42**
	mean of intercept		96.44	96.18	96.26	95.81	95.34	95.06	95.31	95.05	94.43	94.73	93.95	90.45	95.69
		N	37	314	1129	1632	1011	482	255	187	72	31	8	2	5160
**G2**	**75–89**	**ml/min/1.73m** ^**2**^ **/year**	**-1.07**	**-1.07**	**-1.07**	**-1.09**	**-1.08**	**-1.09**	**-1.11**	**-1.12**	**-1.08**	**-1.08**	**-1.07**	**-1.23**	**-1.09**
	mean of intercept		84.09	84.09	83.33	82.88	82.19	81.61	81.61	81.49	80.94	80.36	80.65	80.29	82.29
		N	28	307	1513	2816	2565	1491	1149	817	428	193	62	27	11396
	**60–74**	**ml/min/1.73m** ^**2**^ **/year**	**-0.70**	**-0.65**	**-0.79**	**-0.77**	**-0.80**	**-0.78**	**-0.79**	**-0.78**	**-0.80**	**-0.79**	**-0.79**	**-0.73**	**-0.79**
	mean of intercept		70.12	71.34	71.51	70.82	70.22	69.69	69.26	68.81	68.63	68.53	67.30	67.23	69.61
		N	2	37	246	788	1060	986	968	852	523	275	115	52	5904
**G3a**	**45–59**	**ml/min/1.73m** ^**2**^ **/year**				**-0.48**	**-0.51**	**-0.46**	**-0.52**	**-0.54**	**-0.53**	**-0.60**	**-0.53**	**-0.67**	**-0.53**
	mean of intercept					57.44	57.04	56.90	57.11	56.76	56.47	55.91	56.68	55.57	56.67
		N	0	0	0	10	28	47	65	79	72	45	27	14	387
**G3b**	**30–44**	**ml/min/1.73m** ^**2**^ **/year**												**-0.52**	**-0.52**
	mean of intercept													39.93	39.93
		N	0	0	0	0	0	0	0	0	0	0	0	1	1
**G1-G3b**	**30≤**	**ml/min/1.73m** ^**2**^ **/year**	**-1.32**	**-1.26**	**-1.24**	**-1.19**	**-1.12**	**-1.07**	**-1.01**	**-0.99**	**-0.94**	**-0.91**	**-0.87**	**-0.89**	**-1.11**
	mean of intercept		95.10	92.00	89.73	86.58	83.12	80.16	77.90	76.50	74.63	73.27	70.86	69.79	82.89
		N	82	753	3199	5575	4822	3074	2469	1951	1104	545	212	97	23883

**Fig 3:**
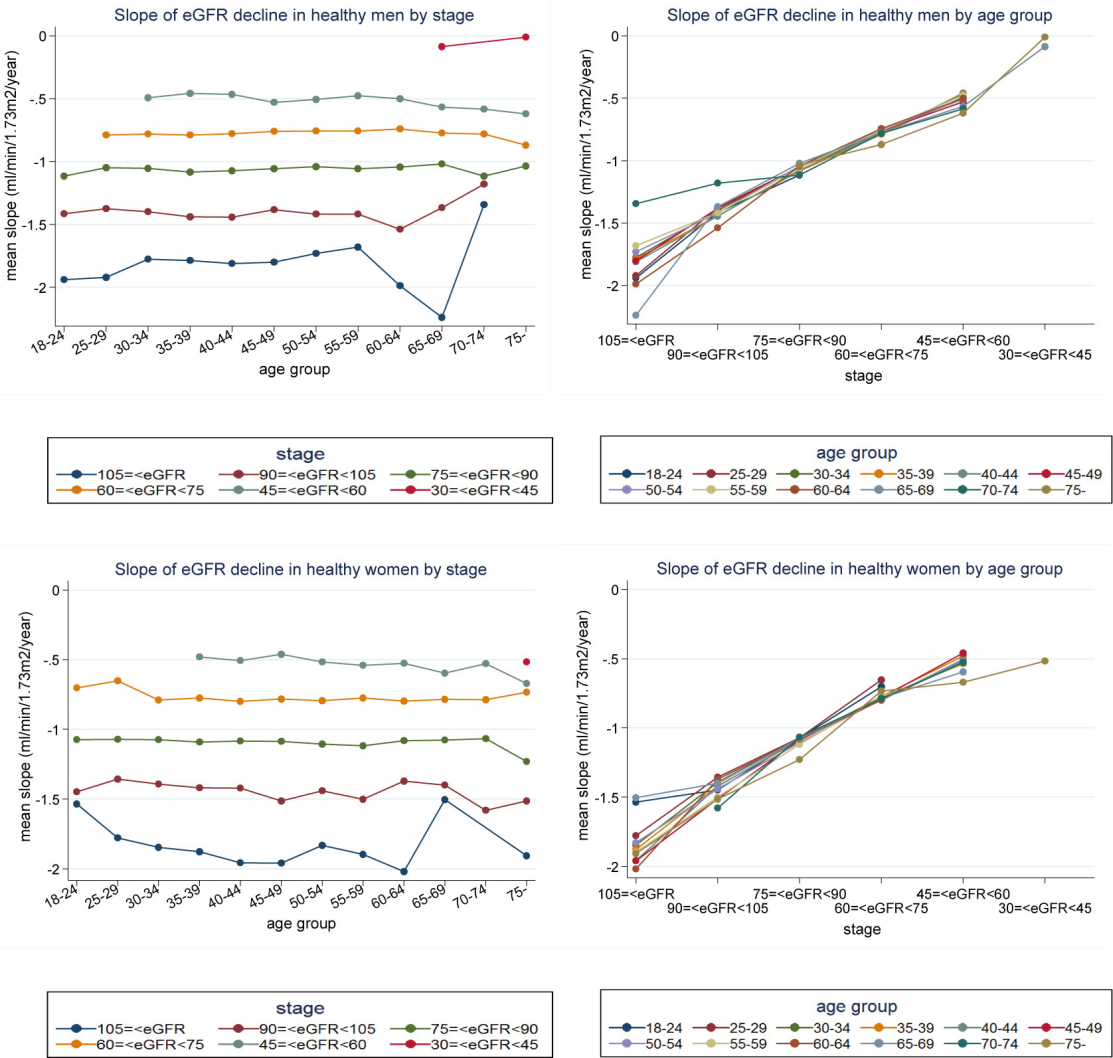
Graphs of the rate of eGFR decline in healthy men and women by renal stage and age group. The mean slope for each stratified component by gender, age and renal stage in Tables 3 and 4 is plotted on the y-axis, and age group and renal stage are plotted on the x-axis. When the slope was stratified by renal stage and gender, all lines ran almost parallel with the x-axis showing age group (left side).When the slope was stratified by age group and gender, all lines almost overlapped (right side). There was little difference in slope within the same renal stage regardless of age, whereas the slope changed depending on the renal stage. The slope was steeper when baseline eGFR was higher (i.e. better renal function) and shallower when baseline eGFR was lower (i.e. renal stage changed from G1 to G3b).

The mean slope for each stratified component in Tables 3 and 4 was plotted on the y-axis, and age group and renal stage were plotted on the x-axis (Fig 3). With stratification by renal stage and gender, all lines ran almost parallel with the x-axis for age group (Fig 3, left side). Thus, there was little difference in the slope of eGFR decline within the same renal stage at all ages. In contrast, with stratification by age group and gender, all lines almost overlapped (Fig 3, right side), indicating that the influence of renal stage on the slope was greater than that of age. Furthermore, the slope was steeper in subjects with better renal function and became shallower as the renal stage advanced (Fig 3).

**Fig 4:**
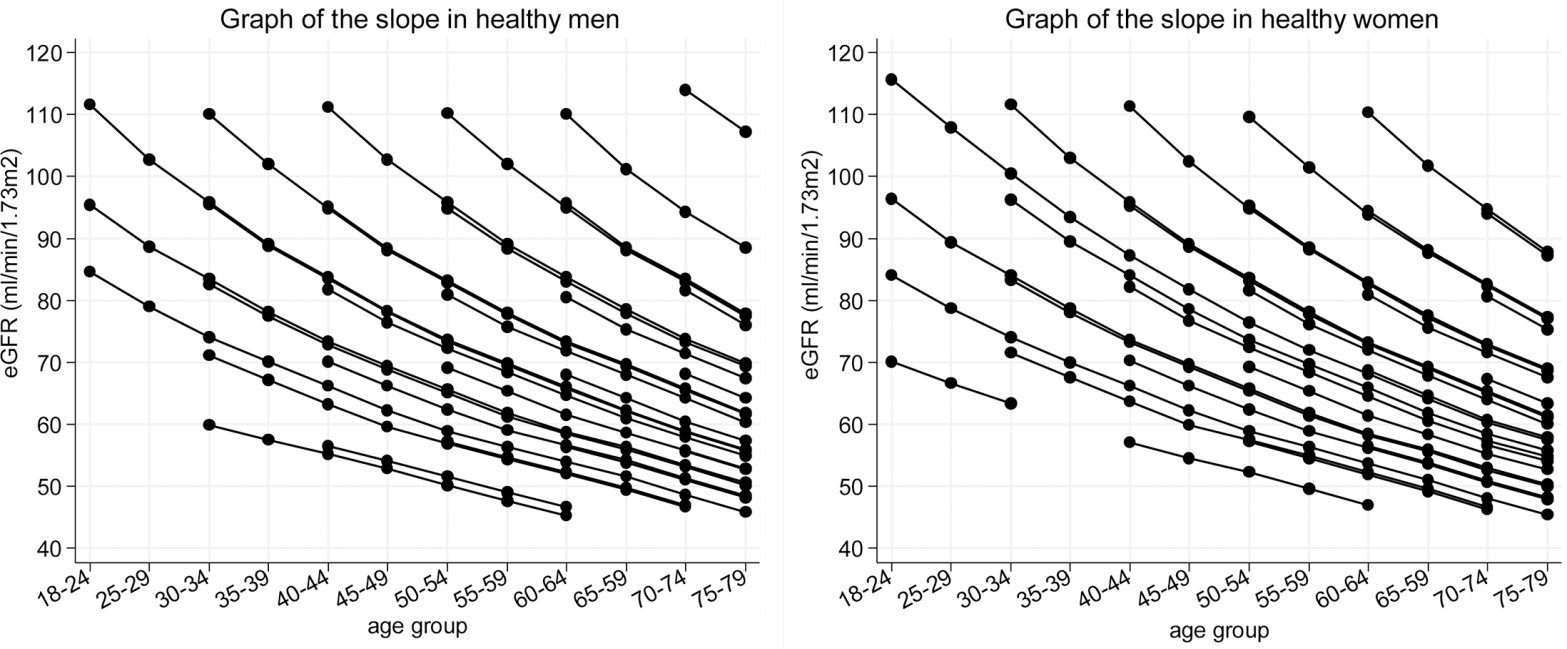
Graph of the slope of eGFR decline (simulated change of eGFR) in healthy men and women. The slope of eGFR decline is depicted as the inclination of each line. The trajectory of renal function based on this slope was predicted with a simulation method. The shape of the graph was gently convex downward, which matches the tendency for the slope of eGFR decline to be steeper for higher baseline eGFR and shallower for lower baseline eGFR.

The slope of eGFR decline was also graphed as the inclination of each line and the trajectory of renal function was predicted by simulation (Fig 4). The shape of the graph was gently convex downward, which matched the tendency for a steeper slope in subjects with higher baseline eGFR and a shallower slope in those with lower baseline eGFR.

### Reference Values for the %slope of eGFR Decline by Gender, Age, and Renal Stage (Longitudinal Study 2)

Reference values were obtained for the %slope of eGFR decline stratified by gender, age, and renal stage in the longitudinal study 2 (S4 Table and S5 Table). Similarly to the slope of eGFR decline, there was little difference in the %slope for different age groups in the same renal stage (S4 Table, S5 Table, and S4 Fig). The %slope was also steeper with higher baseline eGFR and shallower with lower baseline eGFR, although the difference in the %slope among different baseline eGFRs was smaller than that of the slope. To present the distribution of the %slope in each component, the mean %slope±2SD are shown in S4 and S5 Tables. The means (±SD) for the %slope were ‒1.29±0.41%/year in all subjects, ‒1.26±0.39%/year in men, and ‒1.32±0.42%/year in women.

### Factors Related to the Rate of eGFR Decline (Slope and %slope) (Longitudinal Study 3)

An examination of relationships between eGFR decline rate and 15 factors at baseline gave similar results for slope and %slope (Table 5). The slope of eGFR decline was significantly associated with 9 factors: intercept of eGFR, age, gender, urinary protein (15mg/dl; ±), HbA1c, phosphorus, HDL-cholesterol, non-HDL-cholesterol, and BMI (all p<0.0033). The %slope of eGFR decline was significantly associated with 9 factors: intercept of eGFR, age, gender, urinary protein (15mg/dl; ±), uric acid, HbA1c, phosphorus, HDL-cholesterol, and non-HDL-cholesterol (all p<0.0033). Of these factors, the rate of eGFR decline became faster as intercept of eGFR, age, and non-HDL-cholesterol became higher; and became slower as HbA1c, phosphorus, and HDL-cholesterol became higher. The rate of eGFR decline was slower in men than in women, and was also slower in subjects with urinary protein (±) compared to those with urinary protein (‒). The slope of eGFR decline became faster as BMI became higher, and the %slope of eGFR decline became slower as uric acid became higher. The standardized coefficient indicated that eGFR at baseline was the strongest predictor of a future eGFR decline (slope; ‒0.70, %slope; ‒0.41).

**Table 5:** Rate of eGFR decline (Slope and %Slope) evaluated by linear least-squares regression analysis using 15 factors at baseline.

	Slope (ml/min/1.73m^2^/year)						%Slope (%/year)					
Variables	Coefficient	T score	P-value	95% confidential interval		Standardized coefficient	Coefficient	T score	P-value	95% confidential interval		Standardized coefficient
**Intercept of eGFR (ml/min/1.73m** ^**2**^ **)**	**-0.03**	**-175.14**	**<0.001**	**-0.03**	**-0.03**	**-0.70**	**-0.01**	**-83.31**	**<0.001**	**-0.01**	**-0.01**	**-0.41**
**Age(year)**	**-0.003**	**-18.23**	**<0.001**	**-0.004**	**-0.003**	**-0.08**	**-0.003**	**-16.21**	**<0.001**	**-0.004**	**-0.003**	**-0.09**
**Gender**												
**women**	**[reference]**						**[reference]**					
**men**	**0.03**	**5.74**	**<0.001**	**0.02**	**0.04**	**0.03**	**0.03**	**5.10**	**<0.001**	**0.02**	**0.04**	**0.03**
**Urine protein**												
**Negative(-)**	**[reference]**						**[reference]**					
**Trace(15mg/dl;±)**	**0.06**	**6.67**	**<0.001**	**0.04**	**0.07**	**0.02**	**0.07**	**6.73**	**<0.001**	**0.05**	**0.09**	**0.03**
**Uric acid(mg/dl)**	**0.004**	**2.9**	**0.004**	**0.001**	**0.007**	**0.01**	**0.007**	**3.93**	**<0.001**	**0.004**	**0.01**	**0.03**
**HbA1c(%)**	**0.1**	**21.58**	**<0.001**	**0.1**	**0.1**	**0.09**	**0.1**	**22.23**	**<0.001**	**0.1**	**0.1**	**0.1**
**Fasting blood glucose(mg/dl)**	**0.0006**	**2.69**	**0.007**	**0.0002**	**0.001**	**0.01**	**0.0006**	**2.33**	**0.02**	**0.0001**	**0.001**	**0.01**
**Systolic blood pressure(mmHg)**	**0.00006**	**0.42**	**0.7**	**-0.0002**	**0.0003**	**0.002**	**0.00004**	**0.23**	**0.8**	**-0.0003**	**0.0004**	**0.001**
**Serum phosphorus(mg/dl)**	**0.05**	**13.03**	**<0.001**	**0.04**	**0.06**	**0.05**	**0.06**	**13.19**	**<0.001**	**0.05**	**0.07**	**0.06**
**Serum calcium(mg/dl)**	**0.01**	**1.74**	**0.08**	**-0.001**	**0.02**	**0.01**	**0.01**	**2.14**	**0.032**	**0.001**	**0.03**	**0.01**
**HDL-cholesterol(mg/dl)**	**0.001**	**8.79**	**<0.001**	**0.0008**	**0.001**	**0.04**	**0.001**	**8.69**	**<0.001**	**0.001**	**0.002**	**0.05**
**NonHDL-cholesterol(mg/dl)**	**-0.0003**	**-5.25**	**<0.001**	**-0.0004**	**-0.0002**	**-0.02**	**-0.0004**	**-5.53**	**<0.001**	**-0.0005**	**-0.0002**	**-0.03**
**Log Triglycerides(mg/dl)** *	**0.01**	**1.57**	**0.1**	**-0.004**	**0.03**	**0.01**	**0.01**	**1.30**	**0.2**	**-0.01**	**0.04**	**0.01**
**Body mass index**	**-0.002**	**-3.27**	**0.001**	**-0.003**	**-0.0008**	**-0.01**	**-0.002**	**-2.86**	**0.004**	**-0.004**	**-0.0007**	**-0.02**
**Smoking**												
**Never-smoked**	**[reference]**						**[reference]**					
**Ex-smoker**	**0.01**	**2.54**	**0.01**	**0.002**	**0.02**	**0.01**	**0.01**	**2.92**	**0.004**	**0.005**	**0.02**	**0.01**
**Current smoker**	**-0.01**	**-2.24**	**0.025**	**-0.02**	**-0.001**	**-0.009**	**-0.01**	**-2.61**	**0.01**	**-0.02**	**-0.003**	**-0.01**

**Note**: P<0.0033 (0.05/15 = 0.0033) using the Bonferroni correction was considered to be significant.

*The distribution of triglycerides is skewed, and thus the values were log-transformed.

## Discussion

Our longitudinal study revealed for the first time that eGFR decline rate in healthy subjects depended mainly on eGFR at baseline, but not on age. Namely, the rate of eGFR decline was similar in younger and older subjects with similar baseline eGFR values. In other words, the rate of eGFR decline was faster with a higher baseline eGFR and slower with a lower baseline eGFR. In this study all subjects were healthy at baseline, and the longitudinal study indicated the natural course of eGFR decline in healthy people using a large cohort in Japan. These results suggest that healthy subjects are unlikely to progress to the advanced renal stage, such as stages G4 and G5. The reason why the rate of eGFR decline was slower with a lower baseline eGFR is unclear, but a compensatory mechanism might work as kidney function decreases.

In men and women aged 50 years old and over in the cross-sectional study, the 5th percentile of eGFR was <60 ml/min/1.73m^2^, which indicated that 5% of healthy subjects aged ≥50 years old have CKD of stage G3a. If the eGFR of the healthy people between the 2.5th and 97.5th percentiles in our cross-sectional study is considered to be normal, the cutoff value of eGFR at age 50 to 54 years old would be 56 (in men) and 57 ml/min/1.73m^2^ (in women) (S1 Table). Moreover, our longitudinal study suggests that an eGFR decline rate within the mean ±2SD may reflect a process of natural aging in healthy subjects.

The results in our cross-sectional study were similar to those in previous studies of healthy subjects. Wetzel et al. found similar reference values in a population-based prospective cross-sectional study [20, 21] and Okada et al. reported 5th and 95th percentiles of eGFR with almost the same values as those in our study [14]. Approximately similar generation changes of ‒0.51 and ‒0.4 ml/min/1.73m^2^/year were found in the current study and by Wetzel et al. [20], respectively.

Our longitudinal study, however, presented a different finding from previous studies. Lindeman et al. [22] studied the natural rate of eGFR decline in healthy subjects with normal renal function and found that the rate became faster with aging. Imai et al. [15] also found that eGFR decline was faster in older subjects with a lower eGFR in an annual health examination. In contrast, we found that the decline rate became slower with aging, since the baseline eGFR was lower in older subjects. Lindeman et al. [22] included patients with diabetes mellitus, as long as they did not have proteinuria; and Imai et al. [15] included patients with hypertension and/or proteinuria of (1+) and over. In our study, however, we carefully excluded subjects with any abnormal conditions, including diabetes mellitus, hypertension and proteinuria. This may explain the different findings, since the rate of eGFR decline in patients has been reported to be faster than that in healthy subjects. The decline rate in patients with normoalbuminuric type 2 diabetes mellitus with hyperfiltration is significantly faster than that in nondiabetic individuals and became much faster in the later follow-up period [23]. The decline rate in normoalbuminuric type 2 diabetes patients who developed microalbuminuria in the follow-up period was also faster than that in nondiabetic individuals [24]. In the modification of diet in renal disease study [25], the median decline was ‒3.8 ml/min/year for GFR of 25 to 55 ml/min/1.73m^2^; however, in our study in healthy subjects eGFR decline rate was much slower with values of ‒0.52 and ‒0.53 ml/min/1.73m^2^/year in stage G3a in men and women, respectively.

The generation change in the cross-sectional study and the eGFR decline rate in the longitudinal study are different. The cross-sectional study presents the distribution of the initial eGFR of healthy subjects according to the age and depends on the composition of the participants, which may bias the results. In contrast, the longitudinal data represent the trajectory of renal function of each subject and give a true decline rate.

To minimize the effect of a “regression to the mean” in the longitudinal study, the individual intercept of eGFR was used instead of the value of the first eGFR measurement. Furthermore, serial data (2–18 times; median of 4 times, and mean periods of 4.19±2.45 years for men and 4.35±2.47 years for women) were used to calculate the eGFR decline rate using a mixed model analysis. In addition, the sensitivity analyses were performed to investigate the effect of the “regression to the mean”. The individual slope of eGFR decline was recalculated separately in subjects who had only two eGFR measurements (median of 2 times, and mean periods of 1.75±1.37 years for men and 1.79±1.34 years for women) and in those who had 5–18 eGFR measurements (median of 7 times, and mean periods of 6.42±1.51 years for men and 6.54±1.46 years for women) using a mixed model analysis (S6–S9 Tables and S5 Fig). Similarly, the individual slope of eGFR decline was recalculated separately in subjects with a shorter follow-up period (≤2 years; median of 2 measurements, and mean periods of 1.31±0.44 years for men and 1.29±0.41 years for women) and in those with a longer follow-up period (≥4 years; median of 7 measurements, and mean periods of 6.42±1.40 years for men and 6.50±1.38 years for women) (S10–S13 Tables and S6 Fig). In subjects with 2 measurements or with a shorter follow-up period (≤2 years), the SD of the slope was greater especially for the higher intercept of eGFR, and the difference between the slope of stage G1 and that of stage G3a was also greater. Therefore, the “regression to the mean” did seem to have affected the results. However, after the exclusion of subjects with fewer frequency measurements or a shorter follow-up period, the rate of eGFR decline still showed a faster decline with a higher baseline eGFR and a slower decline with a lower baseline eGFR. Additionally, the difference between the rate in subjects with ≥5 measurements (5–18 times; S7 and S9 Tables) and that in all healthy subjects (2–18 times; Table 3 and Table 4) was small, and the difference between the rate in subjects with a longer follow-up period (≥4 years; S11 and S13 Tables) and that in all healthy subjects (2–18 times; Table 3 and Table 4) was also small. Thus we concluded that the “regression to the mean” effect did not cause the tendency in the decline rate and we came to the same conclusion that the rate of eGFR decline was faster with a higher baseline eGFR and slower with a lower baseline eGFR.

There are some limitations in this study. The study was retrospective and the eGFR distribution and rate of decline in healthy subjects should be clarified in a prospective study. The whole study is based on a Japanese population mainly from the Kanto area, where one-third of Japanese people reside. It remains to be determined if the conclusion is applicable to other districts or countries. There were only a small number of subjects with baseline eGFR ≥105 ml/min/1.73m^2^, especially in those aged ≥60 years old, and thus the eGFR decline rate in the higher eGFR group (eGFR ≥105 ml/min/1.73m^2^) may be less reliable. The number of subjects aged ≥70 years old in each stage was small, which made it difficult to evaluate the results in elderly people. The number of subjects with stage G3b was even smaller (n = 2 in men, and n = 1 in women), and there were no subjects with stages G4 and G5 in the study. Therefore, eGFR decline could not be evaluated in stages G3b, G4, and G5. In addition, the reason why the eGFR decline rate was slower with a lower baseline eGFR is unclear and further studies are needed to clarify the underlying mechanisms.

## Conclusions

To the best of our knowledge, this is the first study to determine reference values for the rate of eGFR decline stratified by gender, age, and renal stage in healthy subjects. The rate of eGFR decline depended mainly on baseline eGFR, but not on age. A higher baseline eGFR (better renal function) was related to a faster rate of eGFR decline, and lower baseline eGFR was associated with slower eGFR decline.

## Supporting Information

S1 Fig Total visits of subjects in the longitudinal study. The longitudinal data show that the frequency of creatinine measurements ranged from 2 to 18 times in a row. The median frequency was 4 times for both genders.
(TIF)

S2 Fig Interval (in years) between the first and last visits of subjects in the longitudinal study. The mean interval from the first to the last visit was 4.19 ± 2.45 years for men and 4.35 ± 2.47 years for women.
(TIF)

S3 Fig Generation changes in healthy subjects in the cross-sectional study. Plots show the eGFR of healthy subjects according to age. The change in eGFR with older age (i.e.generation change) was calculated as the coefficient of the fitted line, which was estimated for the relationship between eGFR and age by linear least-squares regression analysis. The generation change was ‒0.51 ml/min/1.73m^2^/year in all subjects, ‒0.46 ml/min/1.73m^2^/year in men, and ‒0.54 ml/min/1.73m^2^/year in women.
(TIF)

S4 Fig %Slope of eGFR decline in healthy men and women by renal stage and age group. The mean %slope in each stratified component by gender, age and renal stage is plotted on the y-axis, and the age group and renal stage are plotted on the x-axis. When the %slope was stratified by renal stage and gender, all lines ran almost parallel with the x-axis for age group (left side).When the %slope was stratified by age group and gender, all lines almost overlapped (right side). Similarly to the slope of eGFR decline, there was little difference in the %slope within the same renal stage regardless of age. The %slope was steeper when the baseline eGFR was higher (i.e. better renal function) and became shallower when baseline eGFR was lower (i.e. advanced renal stage).
(TIF)

S5 Fig Total visits and interval between the first and last visits of subjects with 2 and 5–18 measurements in the longitudinal study. In subjects with fewer measurements (2), the median was 2 times for both genders, and the mean intervals from the first to the last visit were 1.75 ± 1.37 years for men and 1.79 ± 1.34 years for women. For those with more measurements (5–18), the median was 7 times for both genders, and the mean intervals were 6.42 ± 1.51 years for men and 6.54 ± 1.46 years for women.
(TIF)

S6 Fig Total visits and interval between the first and last visits of subjects with shorter (≤2 years) and longer (≥4 years) follow-up periods in the longitudinal study. In subjects with a shorter follow-up period (≤2 years), the median was 2 measurements for both genders, and the mean intervals from the first to the last visit were 1.31 ± 0.44 years for men and 1.29 ± 0.41 years for women. For those with a longer follow-up period (≥4 years), the median was 7 measurements for both genders, and the mean intervals were 6.42 ± 1.40 years for men and 6.50 ± 1.38 years for women.
(TIF)

S1 Table Reference values of eGFR per 5-year age group for healthy men and women in the cross-sectional study. Note: P2.5, P5, P25, P50, P75, P95, and P97.5 are the 2.5th, 5th, 25th, 50th, 75th, 95th and 97.5th percentiles of the reference value of eGFR, respectively.
(XLSX)

S2 Table Slope (ml/min/1.73m^2^/year) of eGFR decline in healthy men. (XLSX)

S3 Table Slope (ml/min/1.73m^2^/year) of eGFR decline in healthy women. (XLSX)

S4 Table %Slope (%/year) of eGFR decline in healthy men. (XLSX)

S5 Table %Slope (%/year) of eGFR decline in healthy women. (XLSX)

S6 Table Slope (ml/min/1.73m^2^/year) of eGFR decline in healthy men with 2 measurements. Note: The statistical analysis in this table is the same as that used in Tables 3 and 4. The mean (±SD) for intercept of eGFR was 81.6±11.1 ml/min/1.73m^2^ in men with 2 measurements.
(XLSX)

S7 Table Slope (ml/min/1.73m^2^/year) of eGFR decline in healthy men with 5–18 measurements. Note: The statistical analysis in this table is the same as that used in Tables 3 and 4. The mean (±SD) for intercept of eGFR was 78.9±11.0 ml/min/1.73m^2^ in men with ≥5 measurements.
(XLSX)

S8 Table Slope (ml/min/1.73m^2^/year) of eGFR decline in healthy women with 2 measurements. Note: The statistical analysis in this table is the same as that used in Tables 3 and 4. The mean (±SD) for intercept of eGFR was 84.7±12.5 ml/min/1.73m^2^ in women with 2 measurements.
(XLSX)

S9 Table Slope (ml/min/1.73m^2^/year) of eGFR decline in healthy women with 5–18 measurements. Note: The statistical analysis in this table is the same as that used in Tables 3 and 4. The mean (±SD) for intercept of eGFR was 81.8±11.7 ml/min/1.73m^2^ in women with ≥5 measurements.
(XLSX)

S10 Table Slope (ml/min/1.73m^2^/year) of eGFR decline in healthy men with shorter follow-up periods (≤2 years). Note: The statistical analysis in this table is the same as that used in Tables 3 and 4. The mean (±SD) for intercept of eGFR was 81.3±11.7 ml/min/1.73m^2^ in men with a shorter follow-up period (≤2 years).
(XLSX)

S11 Table Slope (ml/min/1.73m^2^/year) of eGFR decline in healthy men with longer follow-up periods (≥4 years). Note: The statistical analysis in this table is the same as that used in Tables 3 and 4. The mean (±SD) for intercept of eGFR was 79.7±11.1 ml/min/1.73m^2^ in men with a longer follow-up period (≥4 years).
(XLSX)

S12 Table Slope (ml/min/1.73m^2^/year) of eGFR decline in healthy women with shorter follow-up periods (≤2 years). Note: The statistical analysis in this table is the same as that used in Tables 3 and 4. The mean (±SD) for intercept of eGFR was 84.5±13.1 ml/min/1.73m^2^ in women with a shorter follow-up period (≤2 years).
(XLSX)

S13 Table Slope (ml/min/1.73m^2^/year) of eGFR decline in healthy women with longer follow-up periods (≥4 years). Note: The statistical analysis in this table is the same as that used in Tables 3 and 4. The mean (±SD) for intercept of eGFR was 82.5±11.9 ml/min/1.73m^2^ in women with a longer follow-up period (≥4 years).
(XLSX)
