# Correction: Longitudinal Study of the Decline in Renal Function in Healthy Subjects

**DOI:** 10.1371/journal.pone.0135992

**Published:** 2015-08-13

**Authors:** The PLOS ONE Staff

## Notice of Republication

This article was republished on July 31, 2015, due to the release of confidential or copyrighted material. Please download this article again to view the correct version.
