# Supplementary figures and images for: Longitudinal Study of the Decline in Renal Function in Healthy Subjects
Source: PLoS One. 2015 Jun 10;10(6):e0129036. doi: 10.1371/journal.pone.0129036 (PMC4464887; doi:10.1371/journal.pone.0129036)

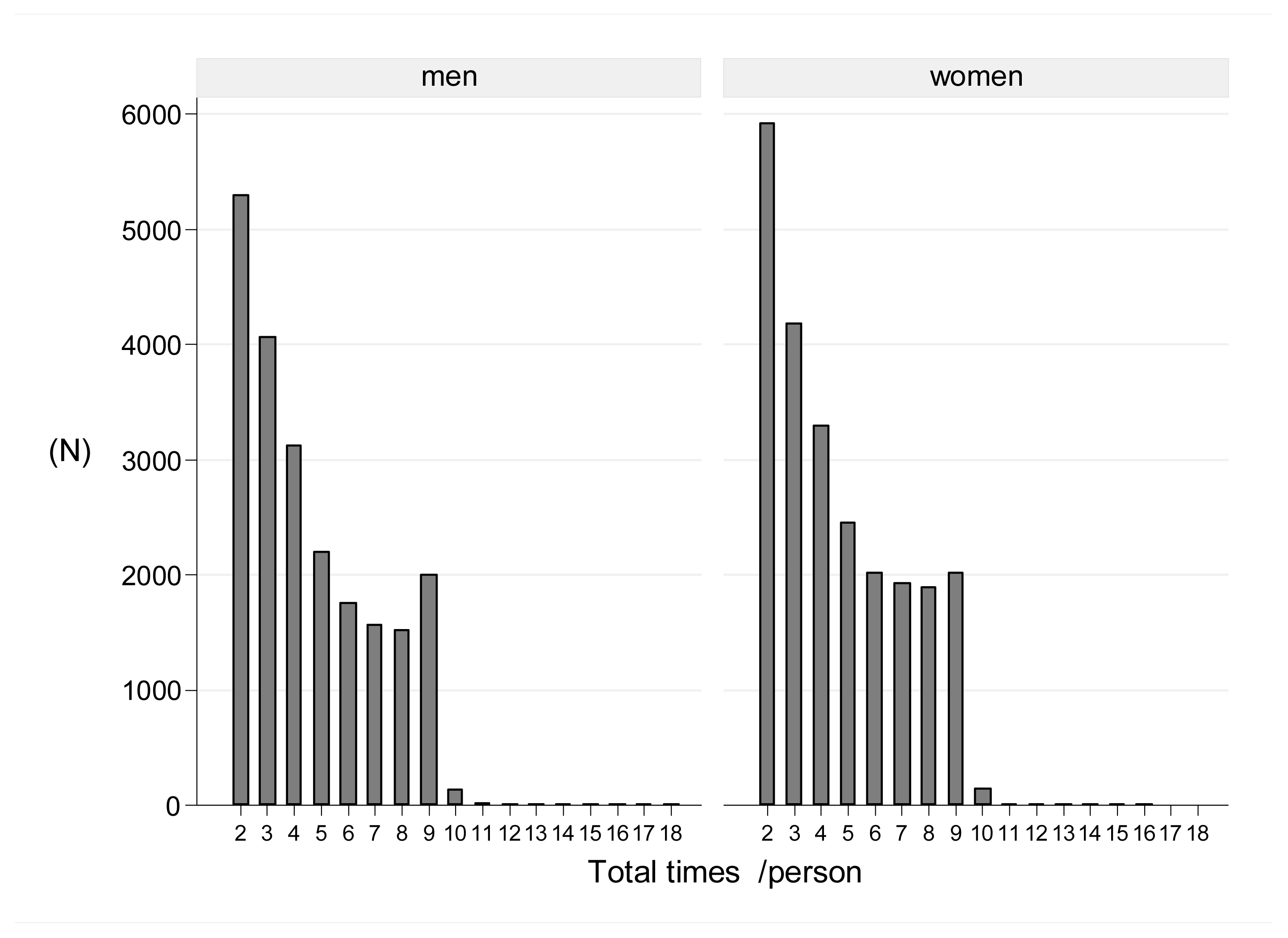

Supplement: S1 Fig — The longitudinal data show that the frequency of creatinine measurements ranged from 2 to 18 times in a row. The median frequency was 4 times for both genders. (TIF) [file pone.0129036.s001.tif]

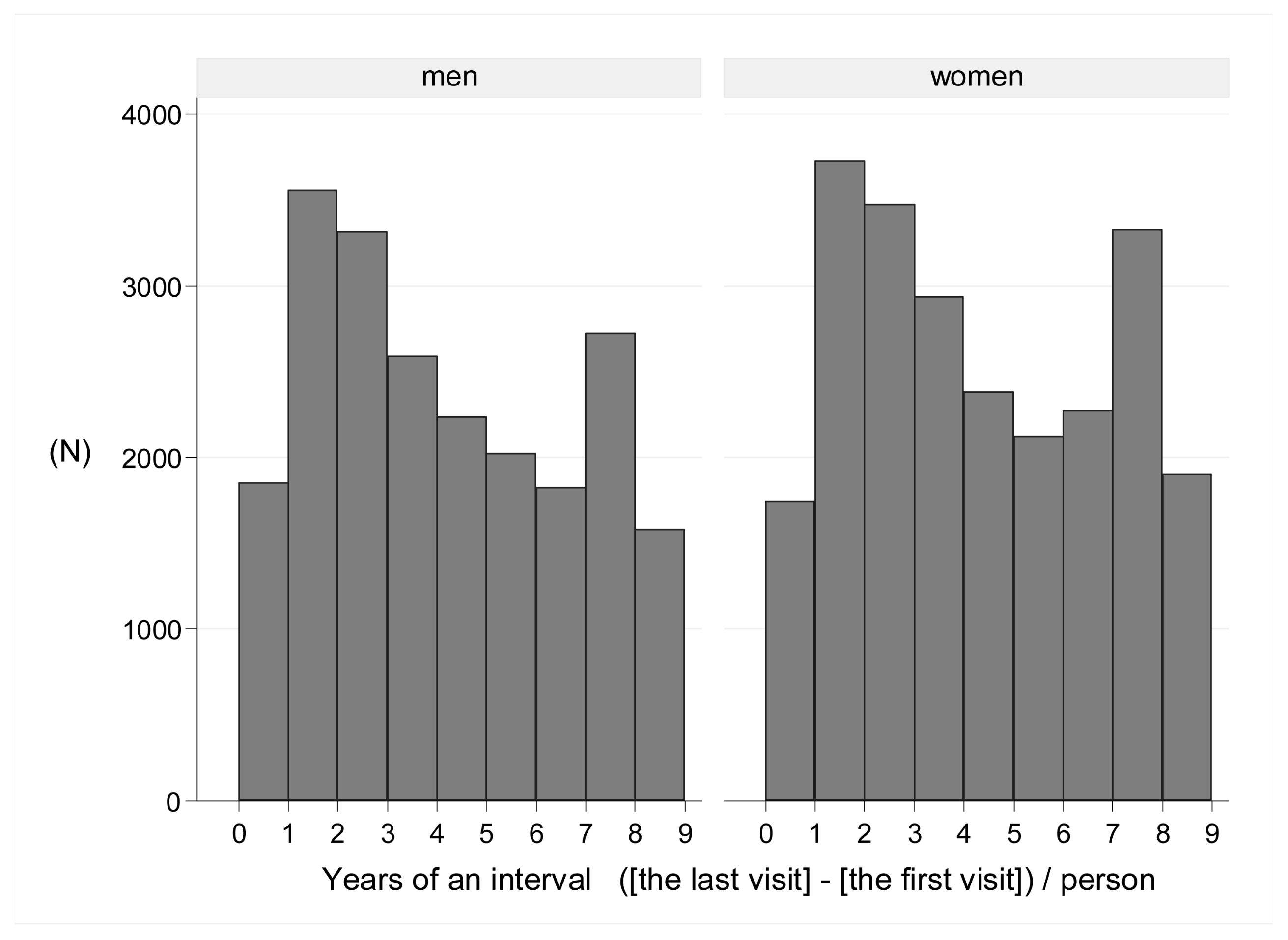

Supplement: S2 Fig — The mean interval from the first to the last visit was 4.19 ± 2.45 years for men and 4.35 ± 2.47 years for women. (TIF) [file pone.0129036.s002.tif]

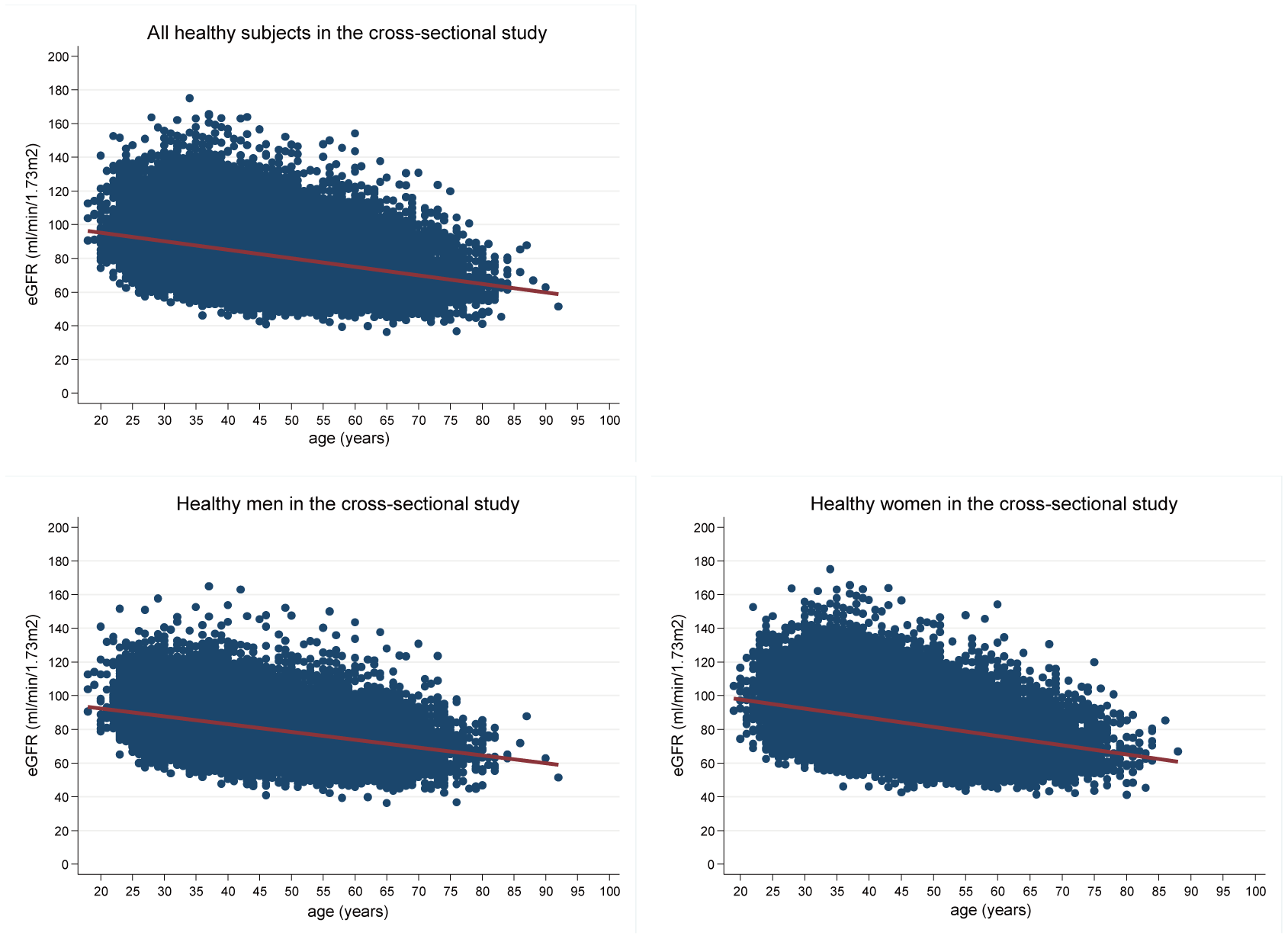

Supplement: S3 Fig — Plots show the eGFR of healthy subjects according to age. The change in eGFR with older age (i.e.generation change) was calculated as the coefficient of the fitted line, which was estimated for the relationship between eGFR and age by linear least-squares regression analysis. The generation change was ‒0.51 ml/min/1.73m2/year in all subjects, ‒0.46 ml/min/1.73m2/year in men, and ‒0.54 ml/min/1.73m2/year in women. (TIF) [file pone.0129036.s003.tif]

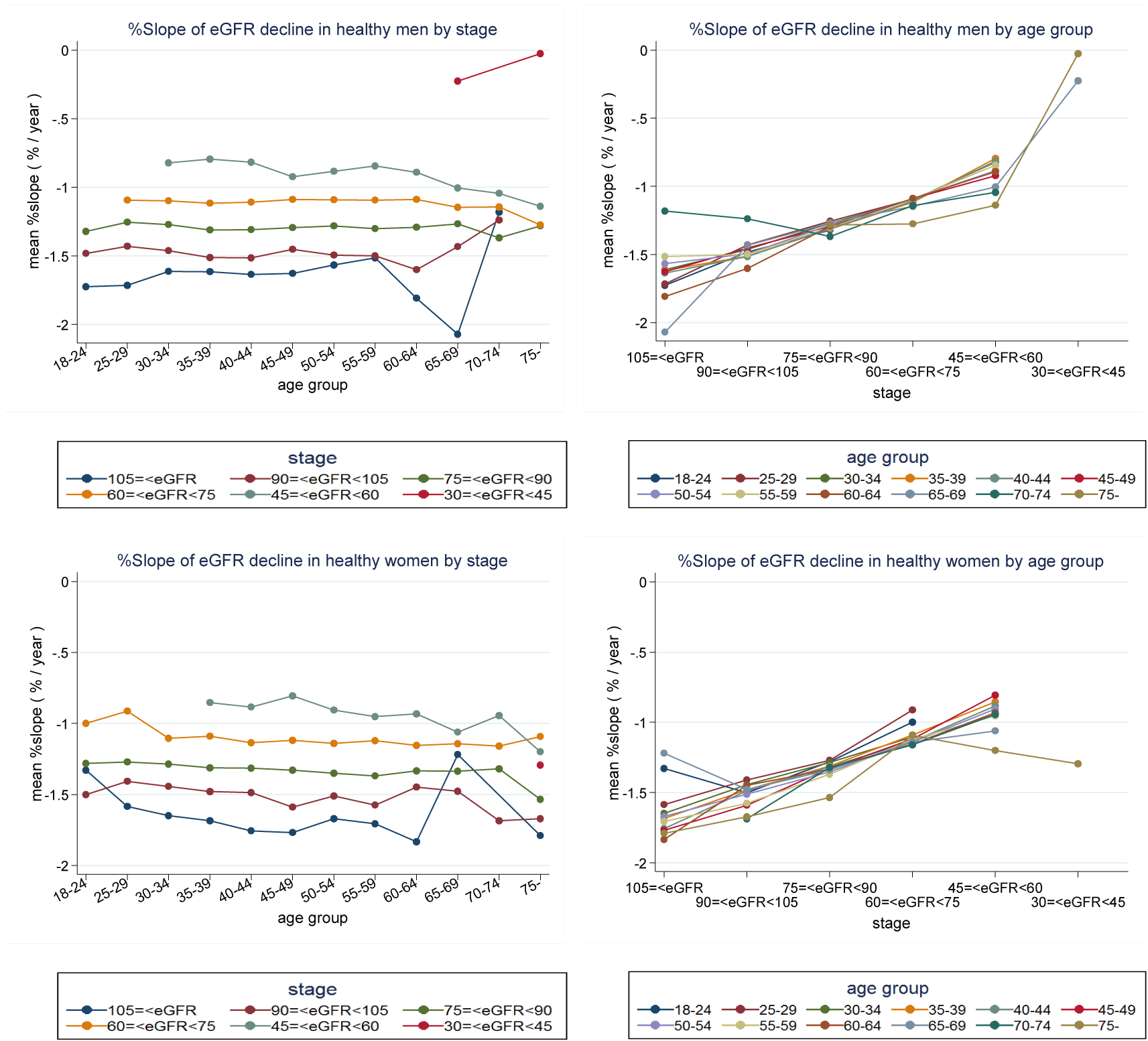

Supplement: S4 Fig — The mean %slope in each stratified component by gender, age and renal stage is plotted on the y-axis, and the age group and renal stage are plotted on the x-axis. When the %slope was stratified by renal stage and gender, all lines ran almost parallel with the x-axis for age group (left side).When the %slope was stratified by age group and gender, all lines almost overlapped (right side). Similarly to the slope of eGFR decline, there was little difference in the %slope within the same renal stage regardless of age. The %slope was steeper when the baseline eGFR was higher (i.e. better renal function) and became shallower when baseline eGFR was lower (i.e. advanced renal stage). (TIF) [file pone.0129036.s004.tif]

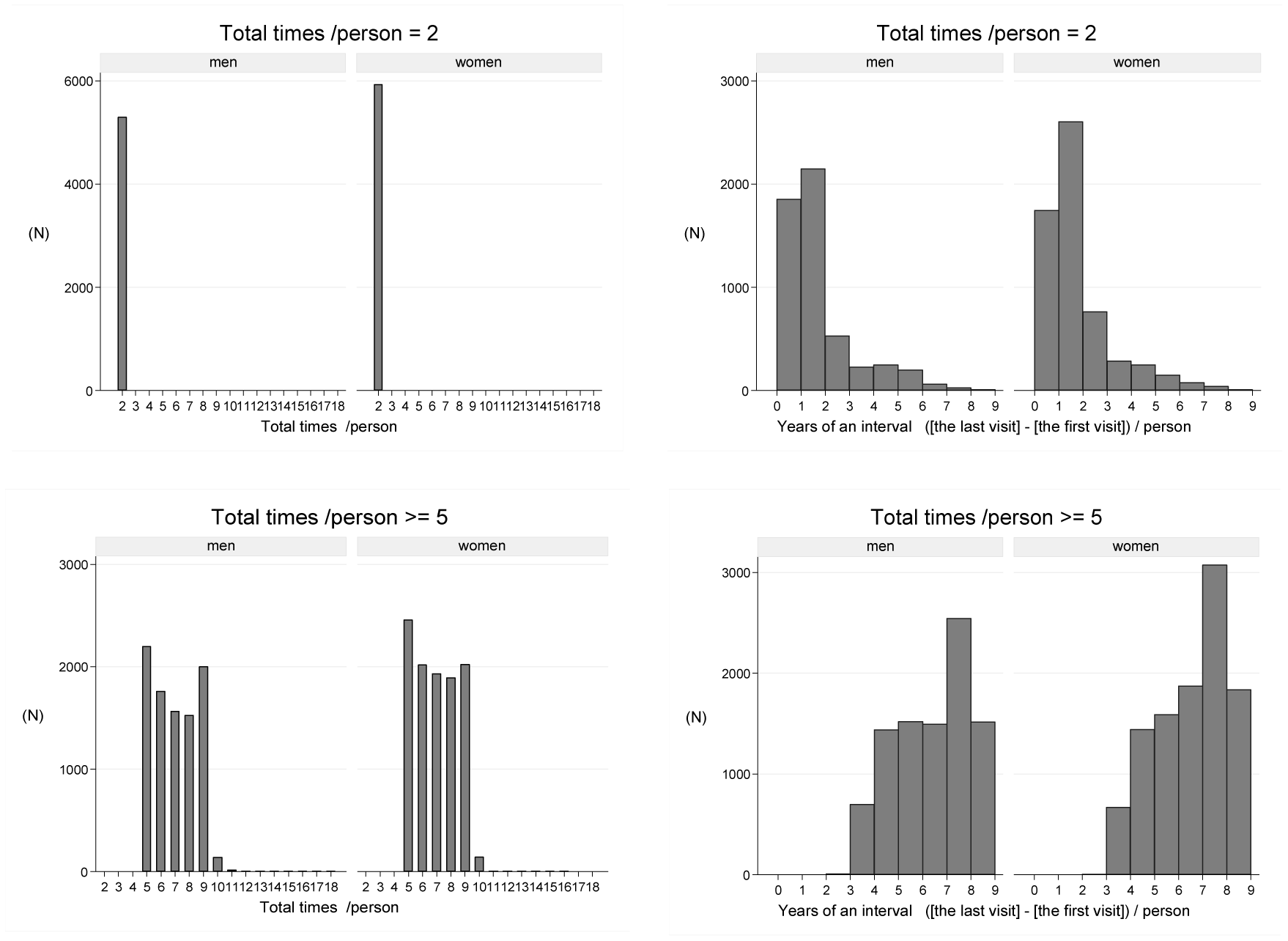

Supplement: S5 Fig — In subjects with fewer measurements (2), the median was 2 times for both genders, and the mean intervals from the first to the last visit were 1.75 ± 1.37 years for men and 1.79 ± 1.34 years for women. For those with more measurements (5–18), the median was 7 times for both genders, and the mean intervals were 6.42 ± 1.51 years for men and 6.54 ± 1.46 years for women. (TIF) [file pone.0129036.s005.tif]

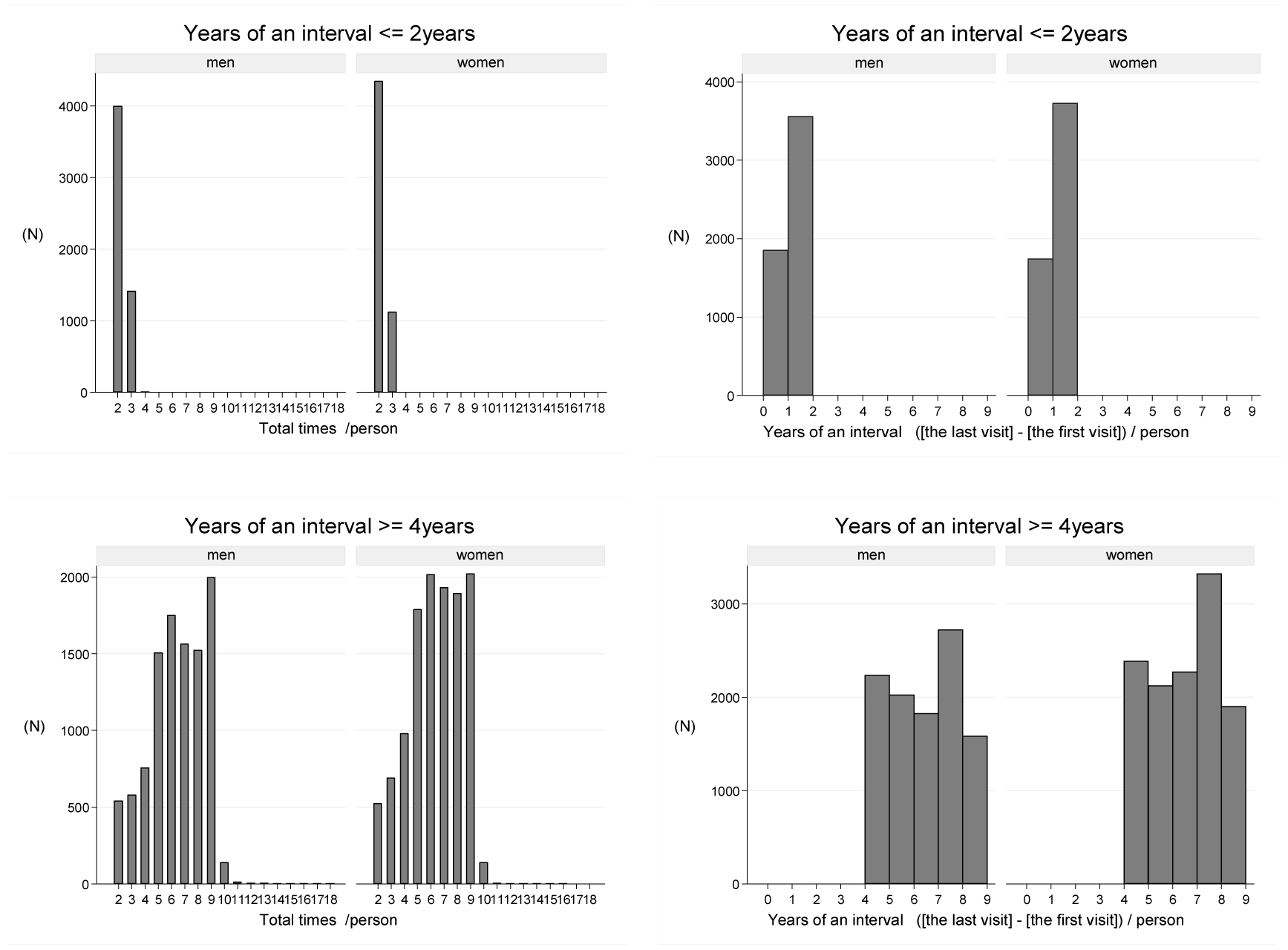

Supplement: S6 Fig — In subjects with a shorter follow-up period (≤2 years), the median was 2 measurements for both genders, and the mean intervals from the first to the last visit were 1.31 ± 0.44 years for men and 1.29 ± 0.41 years for women. For those with a longer follow-up period (≥4 years), the median was 7 measurements for both genders, and the mean intervals were 6.42 ± 1.40 years for men and 6.50 ± 1.38 years for women. (TIF) [file pone.0129036.s006.tif]
